# Revisiting retinoic acid in AML therapy: mechanisms of action and rational combination strategies

**DOI:** 10.1038/s41416-026-03512-z

**Published:** 2026-07-02

**Authors:** Setenay Gupse Özcan, Clara Stanko, Lukasz Szymanski, Martin Fischer, Annamaria Brioli, Arthur Zelent, Tino Schenk

**Affiliations:** 1 Department of Hematology/Oncology, Clinic of Internal Medicine II, Jena University Hospital, Jena, Germany; 2 Institute of Molecular Cell Biology, Center for Molecular Biomedicine (CMB), Jena University Hospital, Jena, Germany; 3 Computational Biology Group, Leibniz Institute on Aging - Fritz Lipmann Institute (FLI), Jena, Germany; 4 Institute of Biochemistry and Cell Biology, Medical Faculty, Otto von Guericke University, Magdeburg, Germany; 5 Department of Molecular Biology, Institute of Genetics and Animal Biotechnology, Polish Academy of Sciences, Magdalenka, Poland; 6 Hematology, Hemostasis, Oncology and Stem Cell Transplantation, Hannover Medical School (MHH), Hanover, Germany

**Keywords:** Acute myeloid leukaemia, Targeted therapies

## Abstract

All-trans retinoic acid (ATRA), a bioactive vitamin A derivative, regulates cellular differentiation by activating retinoic acid receptors (RARs). While ATRA has revolutionised acute promyelocytic leukaemia (APL) treatment, its efficacy in non-APL acute myeloid leukaemia (AML) remains limited due to intrinsic resistance mechanisms, including aberrant epigenetic states and signalling pathways. This review summarises recent mechanistic and translational advances in ATRA-based combination therapies that target differentiation blockade. We focus on the interplay between retinoic acid signalling, chromatin regulation, cell cycle control, and apoptosis, with a particular emphasis on AML. Epigenetic regulators, including the lysine-specific demethylase LSD1 (KDM1A) and the acetyltransferase GCN5 (KAT2A), together with dysregulated cell cycle control and oncogenic signalling, including Fms-like tyrosine kinase 3 (FLT3) and phosphoinositide 3-kinase (PI3K)/protein kinase B (AKT)/mammalian target of rapamycin (mTOR), sustain proliferation and block differentiation. Targeting these pathways with specific inhibitors synergises with ATRA to restore myeloid maturation, induce cell cycle arrest, and promote apoptosis. Future studies should identify predictive biomarkers for patient stratification to translate these synergistic concepts into clinical benefit.

## Introduction

Retinoic acid signalling plays a central role in regulating gene expression programs that determine cell fate decisions across diverse tissues. All-trans retinoic acid (ATRA), also known as tretinoin, is a bioactive derivative of vitamin A and the biologically functional form of retinoic acid (RA). As a ligand for retinoic acid receptors (RARs), ATRA mediates transcriptional programs critical for cell differentiation, proliferation, and development [1–3]. Beyond its physiological role, the antitumor effects of ATRA have been explored across diverse model systems, particularly through differentiation-inducing therapeutic strategies that reprogramme malignant cells toward reduced proliferation and loss of self-renewal. Retinoid-based approaches have shown promise in multiple malignancies, including neuroblastoma [4], breast cancer [5], and pancreatic ductal adenocarcinoma [6].

In previous reviews, the function of ATRA in the treatment of non-promyelocytic acute myeloid leukaemia (non-APL AML) and other malignancies has been discussed, providing insights into aberrant epigenetic states that impair the ability of ATRA to activate its target genes [7, 8]. In this review, we revisit and expand upon the recent mechanisms of ATRA resistance in non-APL AML and provide an overview of rational combination strategies with potential for future therapeutic applications.

## Retinoic acid signalling

**Fig. 1:**
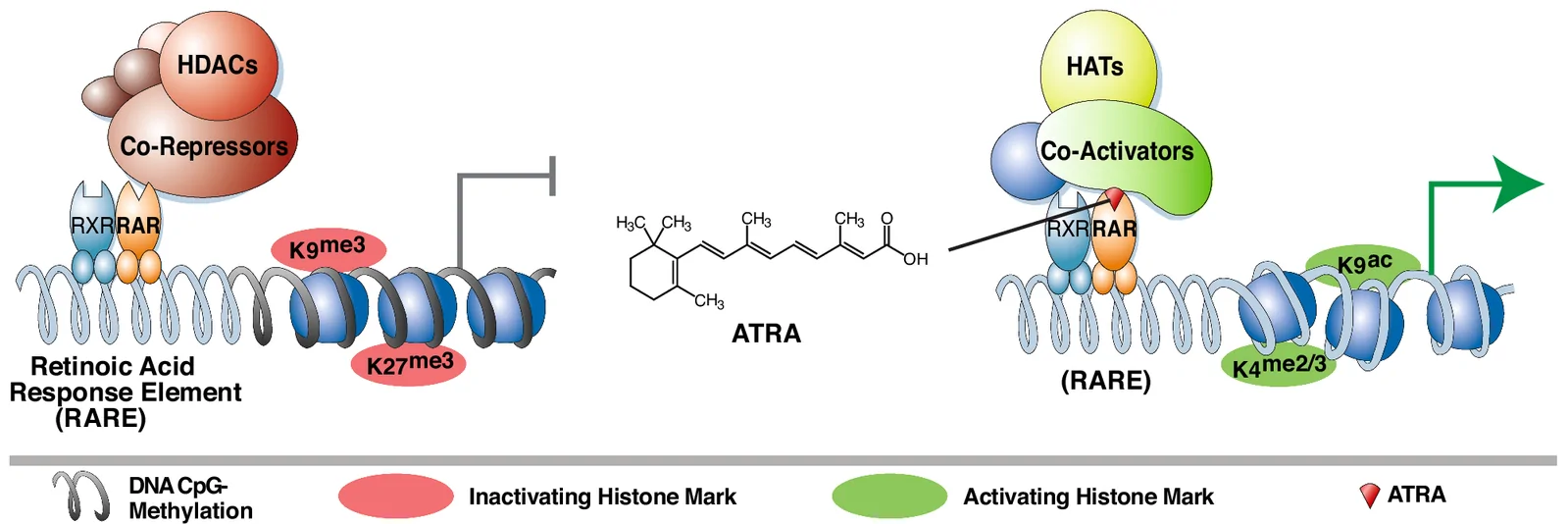
Mechanism of transcriptional activation by all-trans retinoic acid (ATRA) through retinoic acid receptors (RAR). In the absence of ATRA, the heterodimeric nuclear receptor complex composed of retinoic acid receptor (RAR) and retinoid X receptor (RXR) binds to the retinoic acid response element (RARE) within target gene promoters and recruits corepressors and histone deacetylases (HDACs). This repressive complex maintains chromatin in a condensed, transcriptionally inactive state characterised by methylated histone H3 lysine 9 (K9^me3^) and lysine 27 (K27^me3^). Upon ATRA binding, conformational changes in the RAR/RXR complex result in the dissociation of corepressors and the recruitment of coactivators and histone acetyltransferases (HATs). These coactivators catalyse histone acetylation at histone H3 lysine 9 (K9^ac^) and methylation of H3 lysine 4 (K4^me2/3^), leading to chromatin relaxation and transcriptional activation of RA-responsive genes.

Retinoic acid receptors (RARs) are ligand-activated nuclear transcription factors with three subtypes: Alpha, Beta, and Gamma (RARA, RARB, and RARG). By heterodimerizing with retinoid X receptors (RXRs), RARs function as molecular switches to regulate gene expression (Fig. 1). In the absence of RA, RARs form complexes with corepressors such as negative coregulator (NCoR) and silencing mediator for retinoid and thyroid hormone receptors (SMRT), as well as histone deacetylases (HDACs), which modify chromatin to a transcriptionally inactive state. Upon RA binding, the receptor complex undergoes a conformational change, corepressors are released, and coactivators (e.g., CREB-binding protein (CBP)/E1A-associated protein p300 histone acetyltransferases) are recruited. Chromatin is remodelled to an open, transcriptionally active state, enabling the expression of RA-responsive genes [7].

RARA and RARG are particularly important in the differentiation and proliferation of normal hematopoietic and leukaemic cells. While RARG plays a role in maintaining stemness and progenitor-like states, RARA is essential for promoting myeloid differentiation [9, 10]. Although less studied, RARB has been reported to act as a tumour suppressor involved in cellular differentiation, and its dysregulation, including rare fusions in AML, was reported to confer resistance to RA-based therapies [11–13]. Moreover, RA signalling has been shown to be essential for the long-term maintenance and dormancy of functional hematopoietic stem cells (HSCs), protecting them from exhaustion and preserving regenerative capacity [14].

Given their central role in cell differentiation, retinoids have emerged as effective therapeutic agents in cancers characterised by impaired differentiation, where they restore maturation programs; their mechanisms and clinical applications are discussed below.

## Differentiation blockade and therapy in acute myeloid leukaemia

Differentiation is a fundamental biological process where cells progressively acquire structural and functional characteristics that enable them to perform specific roles within a tissue [15]. At its terminal stage, differentiation couples cell cycle exit with the loss of self-renewal capacity, thereby establishing an antiproliferative barrier that suppresses malignant transformation [16]. Accumulating evidence points to disrupted differentiation and cellular plasticity as additional complexities in cancer hallmarks [17].

In AML, mutations in lineage-regulating transcription factors (e.g., *runt-related transcription factor 1 (RUNX1)*, *CCAAT enhancer-binding protein alpha (CEBPA)*), epigenetic regulators (e.g., *DNA methyltransferase 3 alpha (DNMT3A)*, *tet-methylcytosine-dioxygenase 2* (*TET2)*), and fusion oncoproteins (e.g., promyelocytic leukaemia (PML)-RARA) block cell differentiation, leading to aberrant cell proliferation [18, 19]. The concept of differentiation therapy emerged in the 1970s and 1980s, based on several pioneering studies [20–22]. In 1987, Huang et al. demonstrated the first clinical use of differentiation therapy, achieving remission in six APL patients treated with ATRA [23]. Following its initial use in combination with chemotherapy for several decades, ATRA in combination with arsenic trioxide (ATO) has become the standard of care for APL, a leukaemia defined by the fusion of the PML with RARA resulting from the t [15, 17] translocation [24]. Mechanistically, PML-RARA disrupts PML nuclear bodies and reorganises them into microspeckles, thereby impairing checkpoint signalling and repressing retinoic acid-responsive genes through recruitment of histone deacetylases (HDACs) and corepressor complexes [25–27]. These effects can be pharmacologically reversed by ATO, which binds the PML moiety of the fusion protein and triggers its proteasomal degradation, and by ATRA, which restores transcription and induces terminal differentiation [7, 28].

Despite the lack of mutations in RARs, the effect of ATRA on non-APL AML was reported to be limited [7, 28, 29]. Non-APL AML shows heterogeneous differentiation blockade that affects RA-responsive transcriptional programs, often through epigenetic repression of RARA and its downstream targets. Additional RARA-independent mechanisms extend the differentiation block beyond RA-responsive pathways. Recurrent mutations in epigenetic regulators such as DNMT3A, TET2, and isocitrate dehydrogenase 1 and 2 (IDH1/2) alter DNA methylation and hydroxymethylation programs and impair myeloid differentiation [30]. In core-binding factor AML (CBF-AML), fusion oncoproteins such as RUNX1-RUNX1T1 and CBFB-MYH11 repress PU.1/CEBPA-driven differentiation through corepressor and HDAC recruitment, whereas in NPM1-mutant AML, menin-KMT2A-dependent HOXA9/MEIS1 upregulation sustains leukaemic self-renewal [31]. This diverse pathogenesis further complicates differentiation blockade.

Retinoids are physiological inducers of myeloid differentiation; however, in AML, single-agent treatment rarely achieves sustained responses due to epigenetic silencing, aberrant corepressor recruitment, and persistent survival signalling. This mechanistic convergence argues for combination strategies to achieve durable therapeutic outcomes.

## Epigenetic barriers to retinoic acid-induced differentiation

Dysregulation of epigenetic processes, particularly DNA methylation and histone modifications, is associated with malignant transformation and tumour progression. Here, we discuss epigenetic abnormalities responsible for impaired differentiation in AML and outline the potential therapeutic combination strategies.

### Hypomethylating Agents (HMAs)

Silencing of tumour suppressor genes through abnormal DNA methylation in promoter regions is a key epigenetic event implicated in the pathogenesis of myelodysplastic syndrome (MDS) and AML [32]. From the late 2000s, DNA methyltransferase inhibitors (DNMTi), azacitidine and decitabine were introduced into AML treatment [33, 34]. These cytidine analogues become incorporated into DNA during replication, where they covalently trap DNA methyltransferases (DNMTs), resulting in DNMT depletion and DNA hypomethylation. Early in vitro work by Momparler et al. demonstrated that combined treatment with decitabine and RA reduced the clonogenic capacity of HL-60 cells and promoted differentiation [35]. Since then, multiple preclinical studies have confirmed the cooperative effects of ATRA and HMAs [36–38]. In U937 cells, ATAC-seq analysis showed that chromatin accessibility, particularly at retinoic acid response element (RARE) motifs, was increased by both decitabine and ATRA and further enhanced by the combination [37]. Of note, in most AML and MDS cell lines, dual treatment was predominantly reported to enhance cell death rather than differentiation. Consistent with these observations, clinical studies indicate that HMA–ATRA combination regimens do not typically induce differentiation syndrome, while being associated with improved response rates and prolonged progression-free survival in patients with MDS with excess blasts [39].

### Inhibiting LSD1 and GCN5 to overcome ATRA-resistance in AML

We and others have demonstrated that lysine-specific demethylase 1 (LSD1, also known as KDM1A), a histone H3 lysine 4 mono- and dimethyl demethylase, functions as a repressor of ATRA-driven myeloid maturation [29]. Cotreatment with ATRA and the LSD1 inhibitors enhanced myeloid differentiation in AML cell lines and ex vivo patient samples. Moreover, it reduced the engraftment capacity of primary AML cells in mouse xenograft models. These findings, together with independent reports [40], highlighted LSD1 as a therapeutic target in AML and underscored the potential of combining retinoids with agents that modulate aberrant epigenetic states. Since then, several selective LSD1 inhibitors have been explored with ATRA in early-phase clinical studies. A published Phase I/II trial of tranylcypromine plus ATRA in relapsed/refractory AML showed modest activity, with a 20% overall response rate and evidence of myeloid differentiation, but no Phase III follow-up has been reported to date [41–44].

**Fig. 2:**
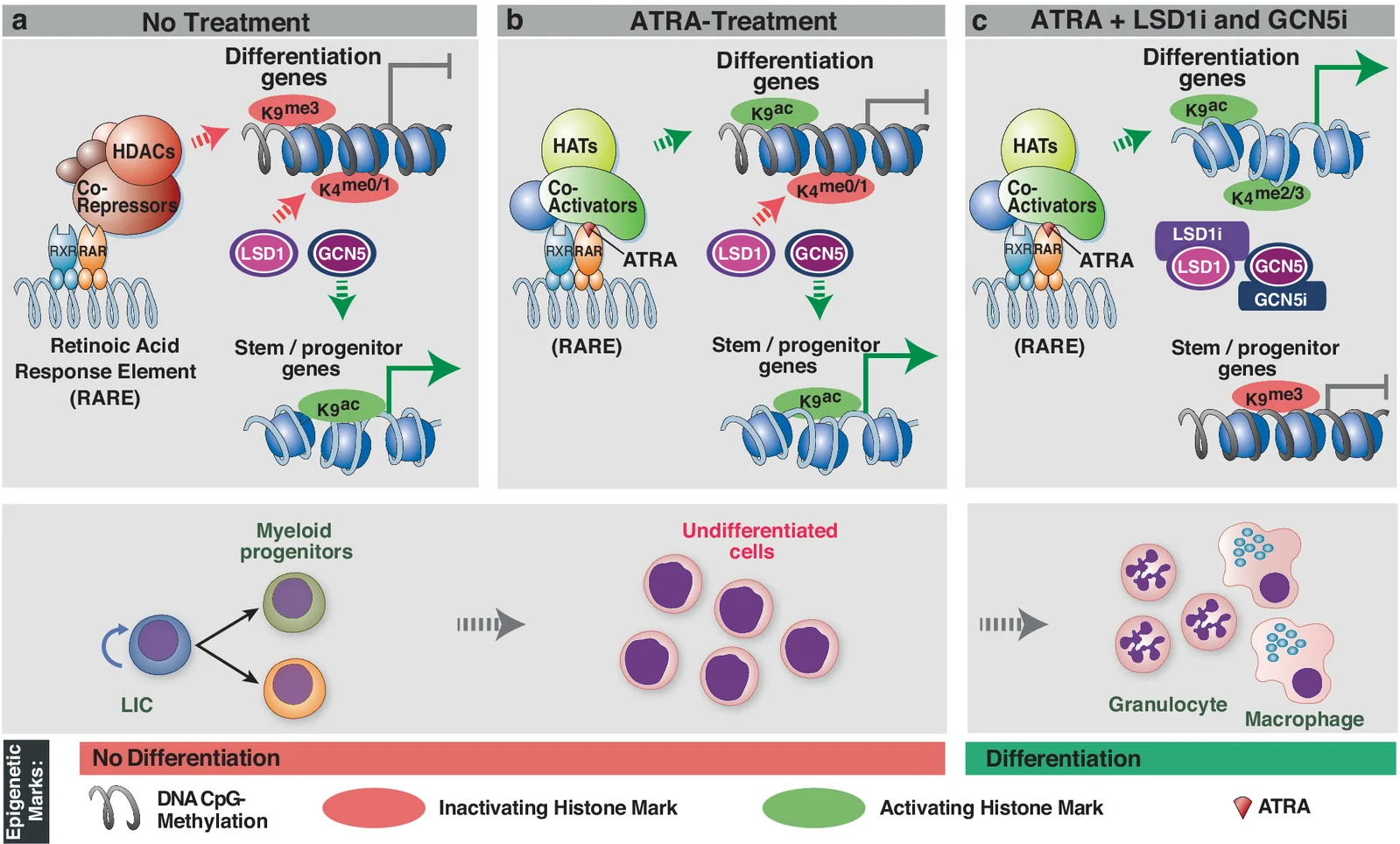
Mechanistic model of ATRA, LSD1, and GCN5 inhibitor combinations in promoting differentiation in acute myeloid leukaemia. **a** In the absence of ligand, retinoic acid receptors (RARs) associate with corepressors and histone deacetylases (HDACs) to maintain transcriptional repression of target genes. Leukaemia-initiating cells (LICs) frequently harbour activating mutations or exhibit aberrant activity of epigenetic regulators such as LSD1 and GCN5, which suppress genes required for myeloid differentiation while sustaining transcription of stem and progenitor cell programs. **b** Treatment with ATRA alone is often insufficient to activate RAR target genes because the chromatin remains locked in an aberrantly repressive state. **c** Combined inhibition of LSD1 (e.g., by tranylcypromine, TCP) and GCN5 (e.g., by MB-3) together with ATRA releases this transcriptional blockade, leading to activation of differentiation-associated genes and repression of stemness-associated transcriptional programs.

Additionally, we identified the histone acetyltransferase GCN5 (also known as KAT2A) as another epigenetic gatekeeper that prevents ATRA-induced differentiation in non‑APL AML [45]. Targeting GCN5 with its inhibitor butyrolactone 3 (MB3) reduced histone H3 lysine 9 acetylation levels, suppressed expression of genes associated with leukaemia stemness, and restored sensitivity to ATRA. Notably, GCN5 inhibition further potentiated the differentiation-promoting effects of LSD1 inhibitor GSK-LSD1. Recent findings have further confirmed these observations, demonstrating that Am80 (tamibarotene), a synthetic RARA-specific retinoid, induces differentiation programs that are highly comparable to those elicited by ATRA in conjunction with LSD1 and GCN5 inhibition (Fig. 2) [46]. Although work on GCN5 inhibition in AML has been limited to in vitro studies, a first-in-human Phase I clinical trial of AUTX-703, a KAT2A/KAT2B degrader, has been initiated in patients with relapsed/refractory AML or MDS and is currently active but not recruiting (Clinicaltrials.gov, NCT06846606).

### Retinoids with menin and DOT1L inhibition

Menin, a nuclear scaffold protein encoded by the *MEN1* gene, is essential for AML development and maintenance of AML with *KMT2A* rearrangement (KMT2Ar) or *NPM1* mutation [47, 48]. As a chromatin adaptor, menin is critical for KMT2A histone methyltransferase complex stability [49], while mutant NPM1 maintains high HOXA9 and MEIS1 expression via the menin-KMT2A complex [48]. Menin inhibitors block this interaction, repressing MEIS1 and HOXA9, and counteracting AML differentiation block [50]. Several inhibitors, including revumenib and ziftomenib, have shown promise in clinical trials, with revumenib receiving FDA approval (Clinicaltrials.gov, NCT04065399, NCT06575296, NCT06222580, NCT06448013, NCT04067336) [51–54]. However, acquired resistance remains a challenge [55, 56]. In 2023, Rausch et al. showed ATRA was highly synergistic with ziftomenib in KMT2Ar and *NPM1* mutated cell lines, inducing differentiation [57]. Another study also found that Am80 and revumenib increased apoptosis and differentiation in cell lines but had modest effects in primary samples [58]. Further clinical studies and high-throughput approaches are needed to unlock the full potential of retinoid-menin inhibitor combinations.

Another epigenetic target in KMT2Ar AML is disruptor of telomeric silencing 1-like (DOT1L), a histone methyltransferase that methylates lysine 79 on histone H3 [59]. DOT1L is crucial for KMT2Ar-driven leukemogenesis via aberrant methylation of *HOXA9* and *MEIS1* loci [60, 61]. Inhibition of DOT1L downregulates HOXA9 and MEIS1, promoting differentiation in cell lines and animal models, suggesting that combination with ATRA could be a promising differentiation-based therapy for KMT2Ar AML. However, pinometostat, a DOT1L inhibitor, showed poor bioavailability in rodents and limited efficacy in a Phase I trial in adult AML patients [62, 63]. As new DOT1L inhibitors are under development with the potential to improve efficacy and pharmacokinetic properties, DOT1L remains an attractive therapeutic target [64].

### Targeting IDH1/2-driven metabolic and epigenetic dysregulation

Isocitrate dehydrogenases (IDHs) are a family of metabolic enzymes that catalyse the conversion of isocitrate to α-ketoglutarate (α-KG) via oxidative decarboxylation. The IDH family consists of three enzymes: IDH1, which is found in the cytoplasm and peroxisomes, and IDH2 and IDH3, which are localised in the mitochondria. IDH3 is primarily involved in the Krebs cycle and is not commonly mutated in cancer, while mutations in IDH1 and IDH2 have been reported in several malignancies, including glioma and AML [65, 66]. Mutations in *IDH1* and *IDH2* impair their normal function to produce α-KG and confer a neomorphic enzymatic function, resulting in the conversion of α-KG to D-2-hydroxyglutarate (2-HG), a known oncometabolite [67, 68].

In AML, *IDH1/2* mutations are found in approximately 20% of patients [69]. In a 2010 study that employed mutational and epigenetic profiling of an AML patient cohort, *IDH1/2*-mutant AMLs displayed global DNA hypermethylation, which altered the function of transcription factors that control myeloid differentiation. Additionally, the authors showed that the activity of TET2, an α-KG-dependent enzyme that catalyses cytosine 5-hydroxymethylation and promotes DNA demethylation, is negatively regulated by 2-HG, resulting in aberrant DNA methylation patterns [30]. A recent study also demonstrated that in *IDH1/2* mutant cancers, 2-HG inhibits KDM5 histone lysine demethylases, which are also myeloid tumour suppressors [70]. Furthermore, through alteration of the epigenetic landscape, 2-HG was also shown to impair DNA damage response by diminishing the chromatin interactions in the DNA damage regions [71].

Since 2017, three IDH inhibitors have been approved by the FDA for the treatment of adult patients with relapsed/refractory AML: enasidenib (IDH2 inhibitor), ivosidenib (IDH1 inhibitor), and olutasidenib (IDH1 inhibitor) [18, 72]. In clinical trials, IDH inhibitors have been shown to exert their therapeutic effect by alleviating the differentiation block, thereby improving blood counts, reducing infection risk, and decreasing the need for transfusion [73–76].

Boutzen et al. reported that the *IDH1* R132H mutation makes AML cells more susceptible to differentiation by ATRA [77]. In HL-60 and MOLM-14 cells, overexpression of IDH1 R132H enhanced the differentiation response to ATRA compared with cells expressing wild-type IDH1, which was further confirmed in primary patient samples harbouring *IDH1* R132H. While mass spectrometry analysis of 2-HG and α-KG confirmed a shift in equilibrium toward 2-HG in *IDH1* mutant cells, treatment with 2-HG sensitised wild-type *IDH1* AML cells to ATRA-induced differentiation. Similarly, IDH2-mutant TF-1 cells showed greater ATRA sensitivity than their wild-type counterparts, and combined enasidenib and ATRA augmented myeloid differentiation marker expression beyond either agent alone in IDH2-mutant cells and patient samples [78]. Further clinical and translational investigations are needed to elucidate the connection between ATRA and *IDH* mutations and to establish whether cotreatment with IDH inhibitors is a viable therapeutic strategy.

## Cell cycle dysregulation as a mechanism that hinders differentiation

Sustained uncontrolled proliferation and evasion of the normal limits of replicative lifespan are hallmarks of cancer [17]. Progression through cell cycle phases is driven by the binding of phase-specific cyclins to cyclin-dependent kinases (CDKs). Cyclin D/CDK4/6 complexes drive cell cycle entry and progression through G1 phase, cyclin E/CDK2 complexes govern S phase entry, cyclin A/CDK2 complexes guide complete DNA replication in S phase, and cyclin B/CDK1 complexes control entry into mitosis [79]. In turn, CDK activity is controlled by cyclin-dependent kinase inhibitors (CKIs) that enable cells to stop cell cycle progression at checkpoints.

In normal hematopoiesis, HSCs remain quiescent, with proliferation initiated only by external cues [80]. Quiescence is a reversible G0 state preserving long-term stem-cell function, while controlled progenitor cell cycle entry is essential for hematopoiesis. In AML, enhanced proliferation of leukaemic blasts coexists with quiescent leukaemic stem cells, persisting in G0 phase and contributing to disease maintenance, relapse, and therapy resistance [81, 82]. This distinction is crucial for differentiation therapy and cell cycle targeting strategies, as effective approaches must address both proliferation and quiescent leukaemia-propagating cells.

Dysregulation of cell cycle through overactive CDKs, loss of CKI function, or aberrant cyclin expression is frequently observed in AML and has been shown to contribute to differentiation blockade and resistance to ATRA treatment [83, 84]. With advances in drug development, cell cycle regulators have become effective targets for overcoming ATRA resistance, opening new horizons in differentiation therapy.

### Targeting cyclin-dependent kinases (CDKs)

In haematological malignancies, CDK6 is frequently overexpressed as a downstream consequence of recurrent chromosomal translocations or upstream oncogenic signalling, driving enhanced proliferation [85–88]. Placke et al. showed that CDK6 plays an essential role in sustaining the leukemogenic activity in AML with *KMT2Ar*, and genetic or pharmacological inhibition of CDK6 can alleviate differentiation block [89]. Another study by Uras et al. revealed an unexpected function of CDK6 beyond its canonical kinase activity, showing that it directly regulates the transcription of *FLT3* and Pim-1 Proto-Oncogene (*PIM1)*, both key mediators of oncogenic transformation [90].

Palbociclib, abemaciclib and ribociclib are ATP-competitive inhibitors of CDK4/6, which have been recently approved by FDA as a therapeutic option for metastatic hormone receptor-positive and human epidermal growth factor receptor 2 (HER2)-negative breast cancer [91]. Despite their efficacy in solid tumours, the use of CDK4/6 inhibitors in non-solid cancers remains largely limited to pre-clinical studies and case reports [92, 93]. Although Phase I/II trials are underway, definitive clinical evidence is still lacking [86]. In our recent work, we have investigated the synergistic potential of palbociclib and ATRA in non-APL AML and found that ATRA and palbociclib combination synergistically promoted differentiation in AML cell lines and exhibited a dose-dependent decrease of cell proliferation, colony-forming capacity and viability [94]. From a mechanistic perspective, the use of this combination is warranted because the G1 phase constitutes the temporal window for cell fate decisions. As a CDK4/6 inhibitor, palbociclib drives cells into G1-arrest through blocking phosphorylation of retinoblastoma protein (RB) and thus activating its repressive binding capacity over E2F transcription factors, thereby allowing ATRA-induced transcriptional regulation to drive lineage-specific gene expression. Consistent with this, transcriptomic analyses revealed that combination treatment induced widespread transcriptional reprogramming in HL-60 cells. While downregulation of E2F1 and CDK1 enforces G1 cell-cycle arrest, key regulators of RA signalling and chromatin remodelling, such as lysine demethylase 6B (KDM6B) and CCAAT enhancer binding protein epsilon (CEBPE), were upregulated, creating conditions that favour initiation of differentiation programs. Further translational and clinical research is needed to dissect the intricate mechanisms of action and to determine which patients may benefit from this therapeutic approach.

## Targeting antiapoptotic pathways in AML

Apoptosis is a form of regulated cell death that is critical for the development and maintenance of tissue homoeostasis, which is primarily executed through two distinct mechanisms: intrinsic and extrinsic pathways [95]. While extrinsic apoptosis is triggered by extracellular proapoptotic signals, such as tumour necrosis factor family ligands, intrinsic apoptosis programs are initiated upon cellular damage, metabolic stress, hypoxia, withdrawal of growth factors or DNA damage [96]. Intrinsic apoptosis is governed largely by B cell lymphoma 2 (BCL-2) protein family, which includes proapoptotic effector proteins (BAX and BAK), proapoptotic BH3-only proteins (BID, BIM, PUMA, Noxa, HRK, BIK, BMF, BAD) and antiapoptotic BCL-2 proteins (BCL-2, BCL-xL, MCL-1, A1, BCL-B, BCL-w) [97]. In AML, antiapoptotic BCL-2 is commonly overexpressed, elevating the signalling threshold required for apoptosis initiation [98, 99].

A potent BCL-2 inhibitor, venetoclax, received accelerated FDA approval in 2018, in combination with an HMA for the treatment of older patients with AML or patients ineligible for intensive chemotherapy [100]. Notably, ATRA was demonstrated to downregulate BCL-2, providing a rationale for studies exploring its potential synergy with venetoclax [101, 102]. Indeed, ATRA in combination with venetoclax has been shown to induce cell cycle arrest and apoptosis in AML cell lines; however, in the study, the cells were treated with an unusually high dose of ATRA (up to 40 μM) based on the IC50 values to induce cell death, which is outside the clinically relevant doses (0.01-1 µM in vitro) [102, 103]. Nonetheless, through transcriptomic analyses, the authors proposed that ATRA helps to reduce venetoclax resistance via downregulation of S100A8 and inhibition of the PI3K/AKT signalling pathway [102].

Another antiapoptotic member of the BCL-2 family is myeloid cell leukaemia 1 (MCL-1), which is known to be critical for the survival of HSCs [104, 105]. Several studies revealed that MCL-1 is indispensable for maintaining the survival and growth of AML cells in mouse models and humans [106, 107]. Intriguingly, in a 2017 study, Wang et al. suggested that MCL-1 serves as a gatekeeper between differentiation and apoptosis. The authors observed that MCL-1 was strongly upregulated on day 2 of ATRA treatment in NB4 cells with PML-RARA fusion, where the ratio of differentiated cells to apoptotic cells was high, whereas by day 6, the ratio equilibrated and MCL-1 levels declined. Concurrently, *MCL-1* knockdown resulted in increased apoptosis of ATRA-treated cells [108]. In recent years, several MCL-1 inhibitors have entered clinical trials, including AZD5991 (NCT03218683), S64315 (NCT02979366) and ABBV-467 (NCT04178902), which showed low response rate and dose-limiting cardiotoxicity [109, 110]. Sorafenib, a multi-kinase inhibitor that has FDA approval for renal cell carcinoma, hepatocellular carcinoma and metastatic differentiated thyroid cancer, was also shown to downregulate MCL-1 and unlock ATRA-induced apoptosis in non-APL AML cell lines and primary AML cells [108, 111]. Taken together, the development of more selective inhibitors targeting antiapoptotic pathways seems to be warranted, and for agents that are already in clinical use, such as sorafenib, clinical studies in combination with ATRA are still lacking.

## Oncogenic signalling

### PI3K/AKT/mTOR pathway inhibition

The PI3K/AKT/mTOR (PAM) pathway has been shown to be essential for maintaining hematopoietic stem cell quiescence, survival, differentiation and metabolic balance, while hyperactivation can lead to leukaemic transformation [112]. Activation of the PAM pathway begins with growth factors or cytokines binding to receptor tyrosine kinases (RTKs), which recruit and activate class I PI3Ks. Activated PI3K in turn catalyses the phosphorylation of phosphatidylinositol-4,5-bisphosphate (PIP2) to phosphatidylinositol-3,4,5-triphosphate (PIP3), leading to recruitment of AKT and its phosphorylation by 3-phosphoinositide-dependent protein kinase 1 (PDK1) and mechanistic target of rapamycin complex 2 (mTORC2). Activated AKT promotes survival by inhibiting proapoptotic proteins, such as BAD and FOXO, while enhancing antiapoptotic effectors, such as MCL-1 and BCL-XL. In parallel, AKT relieves tuberous sclerosis complex 1/2 (TSC1/2), thereby activating mechanistic target of rapamycin complex 1 (mTORC1), which drives protein synthesis through downstream effectors, such as S6 kinase and eukaryotic translation initiation factor 4E-binding protein 1 (4EBP1), driving anabolic metabolism. Together, these signals promote proliferation, metabolic reprogramming, and resistance to apoptosis.

The PAM pathway is aberrantly activated in ~50–80% of AML cases, often due to mutations in receptor tyrosine kinases (e.g., FLT3, c-KIT) or small GTPases (e.g., Ras, Rheb) and is associated with reduced disease-free and overall survival [113–115]. Consequently, it represents a target of therapeutic interest. However, so far, targeting individual components of the pathway showed little to no benefit and unfavourable side effects in clinical trials [116, 117]. Dual mTORC1/2-PI3K inhibitors have demonstrated increased anti-leukaemic activity in preclinical studies, but again, their clinical application was limited by significant adverse effects [118–120]. Optimised combination strategies are required to reach the full therapeutic potential of these agents.

In our previous work, we have tested three small-molecule inhibitors of the PAM pathway in combination with ATRA across various AML cell lines and primary patient samples [121]. We observed that while the PI3K inhibitor ZSTK474 and the mTOR inhibitor WYE132 had a limited effect on cell growth, the dual PI3K/mTOR inhibitor BEZ235 completely suppressed proliferation by driving AML cells into G1 arrest. Moreover, BEZ235 in combination with ATRA synergistically increased expression of the myeloid differentiation marker CD11b and subsequently induced apoptosis. In terms of signalling, we demonstrated that PAM inhibition promoted MYC phosphorylation at Thr58 via glycogen synthase kinase-3 beta (GSK3β), whereas ATRA regulated MYC levels at the transcriptional level. This observation was consistent with the known capacity of ATRA to directly downregulate MYC via an RA-responsive element within the promoter of the *MYC* gene [122]. Moreover, PAM pathway activity has been reported to stabilise MYC by AKT-mediated GSK3β inhibition and mTORC1-driven increased MYC mRNA translation via 4EBP1 and S6K1 [123–125]. The combination of ATRA with PAM inhibitors therefore, represents a promising therapeutic strategy for AML with a strong mechanistic rationale, particularly as synergy may allow dose reduction of PAM inhibitors to mitigate their known toxicity.

### FLT3 signalling

FLT3 is a transmembrane RTK regulating the survival, proliferation, and differentiation of lymphoid and myeloid progenitors [126]. The receptor is activated by its extracellular FLT3 ligand, triggering pathways like PAM, RAS, and signal transducer and activator of transcription 5 (STAT5) [127]. FLT3 mutations occur in about 30% of AML cases, often as internal tandem duplications (FLT3-ITD) (≈25%) [128]. FLT3-ITD mutations cause ligand-independent dimerisation and autophosphorylation, leading to constitutive activation and abnormal STAT5 signalling, driving proliferation and survival [129, 130]. In turn, dysregulated STAT5 activation down-modulates CEBPA and PU.1 [131–133]. Thus, FLT3 and STAT5 are differentiation therapy targets. Recently, several FLT3 inhibitors, including midostaurin, gilteritinib, and quizartinib, have been introduced into clinical practice [134]. For AML patients with FLT3 mutations, midostaurin is now part of first-line therapy, and gilteritinib is used for relapsed or unresponsive patients [18]. Quizartinib, a potent FLT3 inhibitor, was approved by the FDA in 2023. Patients treated with quizartinib showed rapid peripheral blast clearance, with bone marrow samples showing mature neutrophils [135]. Another study showed FLT3 inhibition promoted differentiation of AML cells by downregulating polycomb repressive complex 2 (PRC2) activity [136]. Given the association between FLT3 mutations and differentiation blockade, it is plausible that combining ATRA with FLT3 inhibitors could synergistically prime leukaemic cells for differentiation.

Indeed, early work showed that ATRA enhanced apoptosis induced by the FLT3 inhibitor D-65476 in FLT3-ITD-positive AML cell lines, including MV4-11 and MOLM-13 [137]. Similar synergy was reported with sorafenib, quizartinib, and TTT-3002, and was linked to reversal of FLT3 inhibitor-induced B-cell lymphoma 6 (BCL-6) upregulation, as ATRA reduced BCL-6 expression to baseline, whereas BCL-6 knockdown abrogated the combinatorial effect in FLT3-ITD cell lines [138]. Although in vivo evidence remains limited, xenograft studies further support this interaction: ATRA combined with sorafenib reduced bone marrow leukaemic burden and induced morphologic differentiation in MOLM-14 cells, while ATRA plus quizartinib improved survival and more effectively reduced leukaemic infiltration in the bone marrow and spleen of MOLM-13-bearing mice [138, 139]. Notably, the study by Ma et al. also demonstrated that ATRA enhanced the efficacy of FLT3 inhibition specifically against FLT3-ITD leukaemia stem cells (LSCs), reducing their leukaemia-initiating potential in vivo [138]. Collectively, ATRA and FLT3 inhibitors appear to target distinct but complementary pathways to overcome the differentiation blockade. Although pre-clinical data provide compelling evidence of therapeutic synergy, well-designed clinical trials are needed to evaluate safety and efficacy profiles.

## Discussion

ATRA demonstrates significant therapeutic potential in various cancers, owing to its targeted effects on cell growth and differentiation, alongside minimal toxicity. Clinical studies have consistently shown that retinoid monotherapy produces transient responses, with durable outcomes typically observed only when combined with other therapeutic modalities [28, 140, 141]. Consistent with this, randomised clinical trials in non-APL AML have generally shown limited benefit for adding ATRA to conventional chemotherapy, although more recent studies suggest that activity may depend on treatment backbone and molecular context [141–143]. Moreover, although ATRA is generally well tolerated, its use can be complicated by differentiation syndrome, a potentially life-threatening inflammatory condition characterised by fever, dyspnoea, pulmonary infiltrates, weight gain, effusions, hypotension, renal dysfunction, and leukocytosis [21] These limitations highlight the need for combination strategies that both target survival pathways or epigenetic repression and, in the case of ATRA, enhance therapeutic efficacy to allow lower doses and reduce the risk of differentiation syndrome.

Rather than directly inducing cell death, ATRA promotes terminal differentiation of leukaemic blasts, followed by cell-cycle exit and apoptosis [144]. Differentiation and cell death are distinct biological processes and, in many cases, should be addressed together [108]. For therapies targeting apoptotic machinery, such as venetoclax, the induction of differentiation might represent a double-edged sword, as differentiation may change the cell’s molecular dependencies. In two previous studies employing drug screens, Kuusanmäki et al. demonstrated that the differentiation status of AML cells is a key determinant of venetoclax sensitivity: Immature AML blasts are predominantly BCL-2-dependent, whereas monocytic AML cells shift their survival reliance toward MCL-1. In contrast, AML cells undergoing erythroid or megakaryocytic differentiation primarily depend on the antiapoptotic protein BCL-XL rather than BCL-2 or MCL-1 [145, 146]. Therefore, differentiation direction and status of leukaemic cells should be carefully considered when selecting therapies aimed at inducing apoptosis.

In addition, emerging data suggest that ATRA responsiveness in AML may also be shaped by cell-intrinsic immune activation pathways, including PHD finger protein 8 (PHF8)-associated signalling, as well as by niche-dependent regulation of retinoid availability in the bone marrow microenvironment [147].

The cell cycle represents another attractive target in cancer therapy. Cancer cells often enter the cell cycle more frequently, as many tumours show deregulation of checkpoints and overexpression of cyclins or CDKs. Here, we primarily focused on CDK4/6 inhibitors, as both our research and existing literature indicate strong synergy with ATRA in AML cells. However, it must be emphasised that inhibitors targeting cell cycle represent a broad class of therapeutics beyond CDK4/6 inhibitors, including pan-CDK inhibitors such as flavopiridol and dinaciclib, mitotic kinase inhibitors like aurora kinase and PLK1 inhibitors, as well as several checkpoint kinase inhibitors targeting ataxia telangiectasia and Rad3-related protein (ATR), checkpoint kinase ½ (CHK1/2), or wee-1 like protein kinase (WEE1) [148]. In a comprehensive review, Ruijtenberg and van den Heuvel discussed in detail how temporal coordination between cell cycle exit and differentiation involves mutual antagonism between CDKs and transcription factors that induce tissue-specific gene expression [16]. While preclinical evidence suggests that modulation of the cell cycle can sensitise cells to differentiation in AML, the potential synergy between ATRA and other agents targeting the cell cycle remains largely unexplored and warrants systematic investigation.

In recent years, the therapeutic options for AML have expanded to include agents such as IDH1/2, FLT3, and menin inhibitors. Although these drugs primarily aim to exploit specific genetic vulnerabilities, the induction of differentiation appears to be a major component of their effect, making a combined approach with ATRA plausible. Furthermore, specific subsets of non-APL AML, particularly *IDH1/2* mutant leukaemias, maintain potential ATRA sensitivity, likely due to partial relief of metabolic and epigenetic constraints on myeloid maturation [26, 27]. Additional studies also suggested that in MLL-rearranged AML, ATRA responsiveness was fusion partner dependent, with stronger differentiation in MLL AF9-positive cells [28], while MDS1 and EVI1 complex locus protein (MECOM, also known as EVI1) was reported to modulate the transcriptional and biological response to ATRA [29]. These findings further support that retinoid sensitivity in non-APL AML is context-dependent. Importantly, this context dependence also extends to leukaemic stem cells. Nguyen et al. highlighted that although ATRA may enhance leukaemic stem-cell targeting in some settings, it can in others, such as EVI1-overexpressing MLL-AF9 models, promote stem-cell quiescence and sustain leukaemic stem-cell activity, underscoring the need for genotype-informed combination strategies [28]. This underscores the importance of tailoring treatment decisions to each patient’s genetic profile to achieve optimal therapeutic responses.

**Fig. 3:**
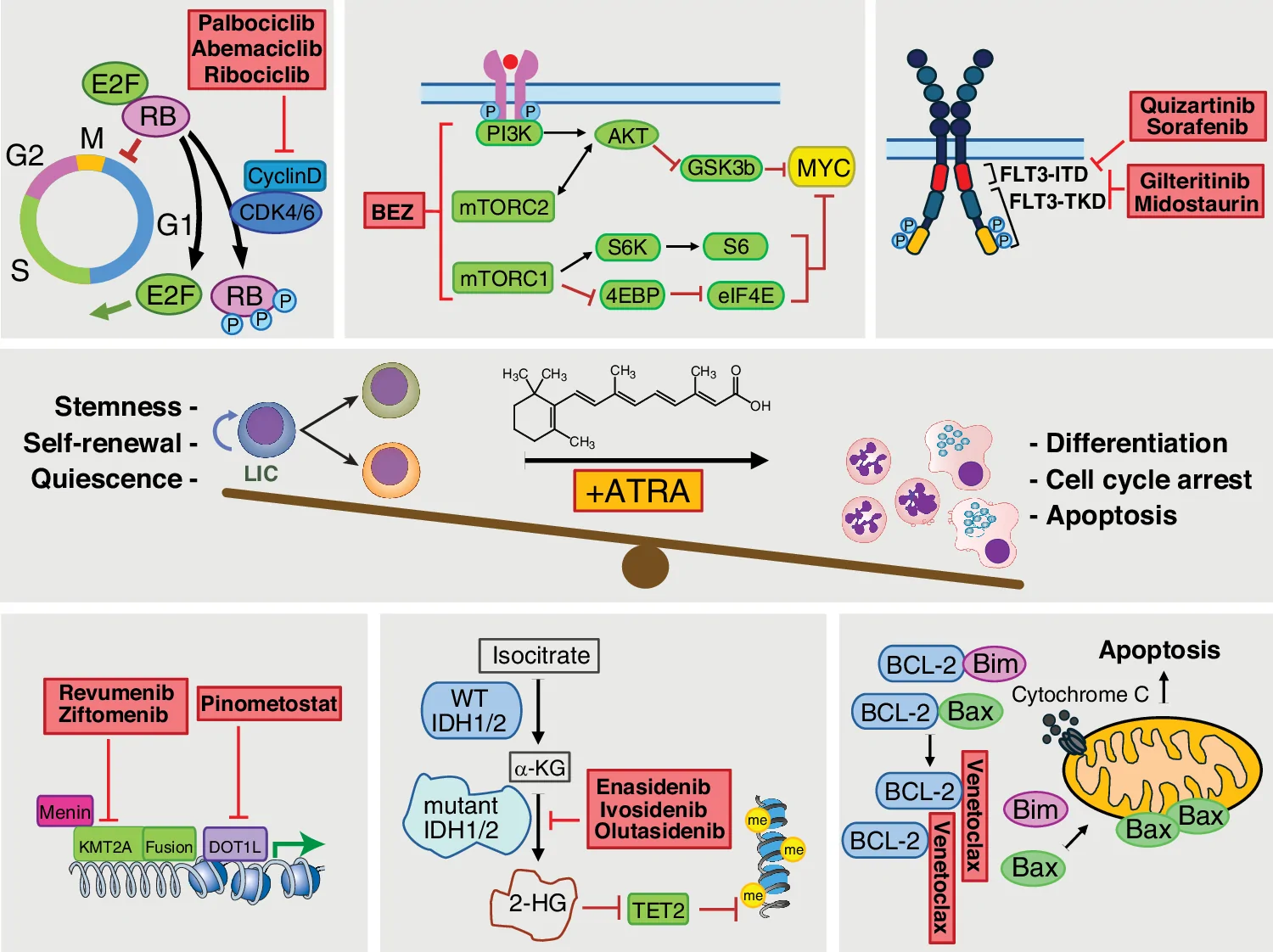
Schematic summary of proposed ATRA-based combination therapies potentially counteracting differentiation blockade in AML. Epigenetic and signalling alterations (middle, left) maintain leukaemic self-renewal and block differentiation. Combined ATRA-based treatments (middle, right) targeting these pathways can potentially restore differentiation, cell-cycle arrest, and apoptosis. Abbreviations: Cyclin-dependent kinase 4/6 (CDK6), retinoblastoma (RB), phosphoinositide 3-kinase (PI3K), mechanistic target of rapamycin complex 1-2 (mTORC1/2), protein kinase B (AKT), glycogen synthase kinase 3-beta (GSK3b), ribosomal protein S6 kinase beta-1 (S6K), eukaryotic translation initiation factor 4E-binding protein 1 (4EBP), eukaryotic translation initiation factor 4E (eIF4E), fms-like protein kinase 3 (FLT3), internal tandem duplicates (ITD), tyrosine kinase domain (TKD), histone-lysine N-methyltransferase 2A (KMT2A), disruptor of telomeric silencing 1-like (DOT1L), isocitrate dehydrogenase 1-2 (IDH1/2), α-ketoglutarate (α-KG), 2-hydroxyglutarate (2-HG), tet methylcytosine dioxygenase 2 (TET2), B-cell lymphoma 2 (BCL-2), Bcl-2-associated X protein (Bax), bcl-2-like protein 11 (Bim).

Given its role as a physiological driver of myeloid differentiation, ATRA represents a rational therapy option, facilitating terminal differentiation and enhancing apoptosis in leukaemic cells that have been primed by targeted therapy. Genetic and epigenetic alterations that shape ATRA responsiveness, as well as retinoid-based combination treatment strategies discussed in this review, are summarised in Fig. 3.

Overall, we aimed to provide a comprehensive perspective on ATRA as a potent mediator of myeloid differentiation and its therapeutic potential beyond APL. Emerging preclinical evidence suggests that the differentiation-inducing effects of ATRA could be extended to additional AML subtypes, particularly when used in combination with targeted agents such as FLT3, IDH, menin, or BCL-2 inhibitors, as well as CDK4/6 inhibitors. However, translating preclinical synergy to the clinic remains challenging, as most ATRA combinations are tested with chemotherapy backbones in which differentiation is not formally assessed. Future studies are needed to optimise combinatorial strategies, define predictive biomarkers, and translate these insights into clinically meaningful regimens, ultimately expanding the therapeutic horizon of differentiation-based approaches in AML and potentially other malignancies.

### Generative AI statement

The author(s) declare that Generative AI tools were used exclusively for grammar and language refinement. No AI tools were used for drafting or interpretation of scientific content.
